# The Silver Lining of COVID‐19: Estimation of Short‐Term Health Impacts Due to Lockdown in the Yangtze River Delta Region, China

**DOI:** 10.1029/2020GH000272

**Published:** 2020-09-01

**Authors:** Ling Huang, Ziyi Liu, Hongli Li, Yangjun Wang, Yumin Li, Yonghui Zhu, Maggie Chel Gee Ooi, Jing An, Yu Shang, Dongping Zhang, Andy Chan, Li Li

**Affiliations:** ^1^ School of Environmental and Chemical Engineering Shanghai University Shanghai China; ^2^ Key Laboratory of Organic Compound Pollution Control Engineering (MOE) Shanghai University Shanghai China; ^3^ SILC Business School Shanghai University Shanghai China; ^4^ Department of Civil Engineering University of Nottingham Malaysia Semenyih Selangor Malaysia; ^5^ Institute of Climate Change (IPI), National University of Malaysia (UKM) Bangi Selangor Malaysia

**Keywords:** premature mortality, COVID‐19, PM_2.5_, Yangtze River Delta

## Abstract

The outbreak of COVID‐19 in China has led to massive lockdowns in order to reduce the spread of the epidemic and control human‐to‐human transmission. Subsequent reductions in various anthropogenic activities have led to improved air quality during the lockdown. In this study, we apply a widely used exposure‐response function to estimate the short‐term health impacts associated with PM_2.5_ changes over the Yangtze River Delta (YRD) region due to COVID‐19 lockdown. Concentrations of PM_2.5_ during lockdown period reduced by 22.9% to 54.0% compared to pre‐lockdown level. Estimated PM_2.5_‐related daily premature mortality during lockdown period is 895 (95% confidential interval: 637–1,081), which is 43.3% lower than pre‐lockdown period and 46.5% lower compared with averages of 2017–2019. According to our calculation, total number of avoided premature death aassociated with PM_2.5_ reduction during the lockdown is estimated to be 42.4 thousand over the YRD region, with Shanghai, Wenzhou, Suzhou (Jiangsu province), Nanjing, and Nantong being the top five cities with largest health benefits. Avoided premature mortality is mostly contributed by reduced death associated with stroke (16.9 thousand, accounting for 40.0%), ischemic heart disease (14.0 thousand, 33.2%), and chronic obstructive pulmonary disease (7.6 thousand, 18.0%). Our calculations do not support or advocate any idea that pandemics produce a positive note to community health. We simply present health benefits from air pollution improvement due to large emission reductions from lowered human and industrial activities. Our results show that continuous efforts to improve air quality are essential to protect public health, especially over city‐clusters with dense population.

## Key Points

- PM_2.5_ concentration decreased substantially during COVID‐19 lockdown in the Yangtze River Delta Region, China
- Number of PM_2.5_‐related premature death decreased due to lockdown
- Avoided premature mortality is mostly contributed by reduced death associated with stroke, ischemic heart disease, and chronic obstructive pulmonary disease

## 1 Introduction

The outbreak of the tragic Coronavirus disease 2019 (COVID‐19) by the end of 2019 has caused tremendous impacts on people's life around the world. At the time of this writing (6th May 2020), COVID‐19 has made more than 3.6 million people sick and led to more than 257,301 deaths worldwide (https://www.statista.com/statistics/, last access on 6th May 2020). During its peak, the pandemic at one point caused over 15,000 new confirmed cases in China in just one single day back in February, and presently in May very few new local infections are reported in China (http://www.nhc.gov.cn/, last access on 6th May 2020). The effective containment of COVID‐19 within China is mostly attributed to a series of prevention and control measures implemented rapidly by the Chinese government. Starting from late January 2020, national emergency response policies were launched in China in order to reduce the intensity of the spread of the epidemic to slow down the increase of number of new cases, including but not limited to: schools shut down, traffic strictly restricted, industries and construction activities suspended, mass gatherings and events canceled or suspended, and social distancing become the new norm. As a result of the massive lockdown, emissions of primary air pollutants from various human and industrial activities decreased substantially and PM_2.5_ concentrations during COVID‐19 lockdown in China has been shown to be much better than previous years during the same period (NASA, 2020; P. Wang et al., 2020). It is well known that poor air quality, with PM_2.5_ (particulate matters with aerodynamic diameters less than 2.5 μm) being a key criteria pollutant, could have adverse health impacts (Boldo et al., 2011; Cao et al., 2012; Song et al., 2017) and lead to premature mortality (Fang et al., 2016; Liu et al., 2016; Lu, Zhou, et al., 2015). An integrated exposure risk (IER) model for PM_2.5_ exposure‐response function is widely used to estimate the premature mortality attributed to PM_2.5_ exposure. For example, Maji et al. (2018) estimates PM_2.5_‐related long‐term premature mortality for 161 cities in China for year 2015 as well as the potential health benefits of air pollution control policies for year 2020. Y. Wang et al. (2020) calculate the number of premature death due to acute and chronic exposure of ambient PM_2.5_ in China during 2013–2017. With substantial reductions in PM_2.5_ concentrations due to COVID‐19 lockdown, a follow‐up question is what are the health impacts of the short‐term changes in air quality.

The Yangtze River Delta (YRD) region is one of the most economic developed and populated regions in China. In the past, the YRD region has frequently experienced heavy haze pollution (Cheng et al., 2013; Wang et al., 2015). With various control strategies continuously being carried out, the overall air quality over the YRD region has greatly improved for the past few years (Ministry of Ecology and Environment of China, 2019). According to the latest report released by the Ministry of Ecology and Environment of China (2020), 40 out of 41 cities in the YRD region has successfully met the goals of reducing PM_2.5_ concentrations during the 2019–2020 fall and winter season.

In our most recent study (L. Li et al., 2020), we investigate the air quality changes over the YRD region due to lowered human activities during COVID‐19 lockdown using multipollutant observations and photochemical model simulations. In this follow‐up study, we attempt to quantify the short‐term health impacts associated with PM_2.5_ changes over the YRD region due to COVID‐19 lockdown. We estimate the premature mortality associated with PM_2.5_ exposure before lockdown and during lockdown periods. Utilizing simulated results based on an integrated meteorology and air quality modeling system, we estimate the number of avoided premature death due to lowered PM_2.5_ concentrations during COVID‐19 lockdown over the YRD region. Methods and results from our previous study (L. Li et al., 2020) are partially adopted in this study to support health related estimation.

## 2 Materials and Methods

### 2.1 Quantitative Analysis PM_2.5_ Changes Due to COVID‐19 Lockdown

**Figure 1:**
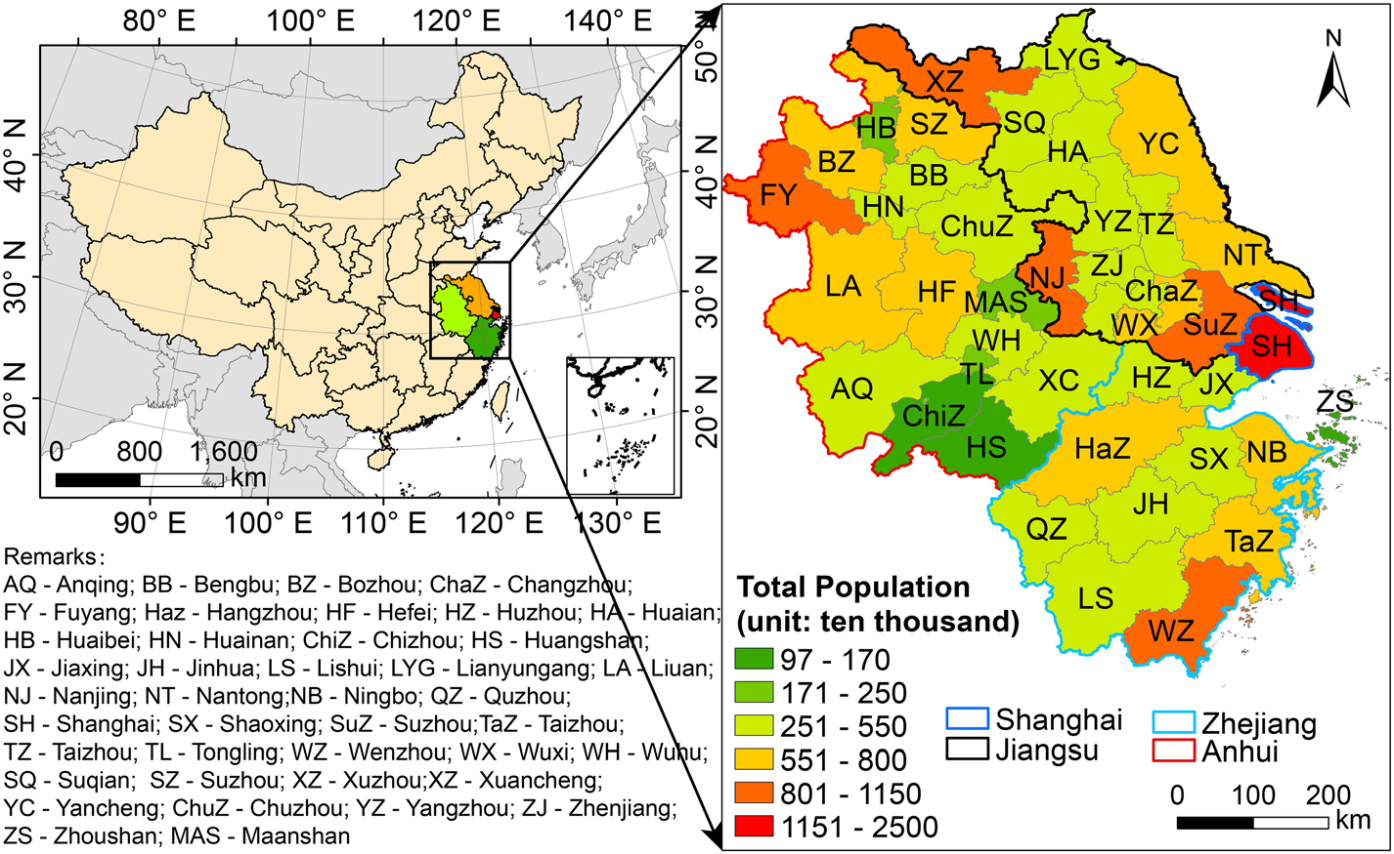
Location of the Yangtze River Delta (YRD) region with city‐level population.

The YRD region, consisting of Shanghai, Jiangsu, Zhejiang, and Anhui province (Figure 1), is one of the most economic developed and populated regions in China. On 23rd January 2020, being one of three earliest provinces (the other two being Hunan and Guangdong provinces), Zhejiang province (located in south of the YRD region) announced provincial lockdown as “Level I” (particularly serious) response, followed by Shanghai and Anhui province on the next day and Jiangsu province 2 days later. Coincided with the Chinese Spring Festival (24th January to 1st February 2020), all kinds of human activities were greatly reduced during Level I response period. With the epidemic gradually controlled, emergency response in Anhui and Jiangsu province was downgraded to Level II (serious) on 25th February, followed by Zhejiang province on 2nd March. Shanghai announced Level II response on 24th March due to high numbers of imported infectious cases. Same as previous study, we define pre‐lockdown period as 1st January to 23rd January, Level I response period as 24th January to 25th February, and Level II response period as 26th February to 31st March.

To quantify the changes of air quality caused by reduced human activities during COVID‐19 lockdown, the integrated Weather Research Forecasting model (WRF)‐Comprehensive Air Quality Model with Extensions (CAMx) modeling system is used (L. Li et al., 2020). Details of model configurations and input data can be found in L. Li et al. (2020) and are briefly summarized here. The integrated WRF/CAMx model is applied to simulate air quality over the YRD region (Figure 1) during pre‐lockdown, Level I response, and Level II response periods. Two parallel simulations are conducted with two sets of anthropogenic emissions while keeping all other inputs and model configurations identical. For the base case simulation, the baseline emissions (i.e., emissions from normal activities assuming no lockdown) are used. For the COVID‐19 scenario, emissions estimated based on reduced human activities due to lockdown are applied. For emission reductions outside the YRD region during lockdown, we applied the reduction ratio used by P. Wang et al. (2020). The relative improvement factor (RF) is defined as the ratio of simulated concentrations between the two scenarios and is applied to the observed concentrations to obtain the theoretical concentrations of air pollutants that would be if there is no lockdown. Results of model performance evaluation of the COVID‐19 scenario show acceptable agreement between simulated and observed results (L. Li et al., 2020).

### 2.2 Premature Mortality Due to Short‐Term PM_2.5_ Exposure

We estimate the premature mortality due to ambient PM_2.5_ exposure based on a widely used log‐linear exposure‐response function below (Fang et al., 2016; Gao et al., 2016):

(1) $$Y=\sum_{k}P\times \left(1-e^{-\beta_{k}\left(C-C_{0}\right)}\right)\times R_{k},$$

where *Y* is the number of premature deaths caused by ambient PM_2.5_ exposure due to five leading causes (*k* = 5): cerebrovascular disease (stroke), ischemic heart disease (IHD), chronic obstructive pulmonary disease (COPD), lung cancer (LC) for adults (≥25 years), and acute lower respiratory infection (ALRI) for infants (<5 years). *β* is the cause‐specific exposure‐response coefficients and values reported from a meta‐analysis study (Lu, Xu, et al., 2015) are utilized in this study. For an increase of 10 μg/m^3^ PM_2.5_, *β* is 0.63% [95% confidential interval (CI): 0.35–0.9%] for cardiovascular disease (i.e., stoke, IHD) and 0.75% (95% CI: 0.39–1.11%) for respiratory disease (i.e., COPD, ALRI, LC). The baseline incidence rate (*R*) at provincial level is obtained from the Sixth National Population Census (http://www.stats.gov.cn/tjsj/pcsj/rkpc/6rp/indexch.htm, last access on 20th April 2020) and the contribution of individual disease to total mortality are based on the national estimates from the Global Burden of Diseases (GBD) project of Institute for Health Metrics and Evaluation (IHME) and Health Effects Institute (HEI) for year 2017 (https://vizhub.healthdata.org/gbd-compare/, last access on 23rd April 2020). According to GBD study, stroke, IHD, COPD, LC, and ALRI contribute 20.2%, 16.7%, 9.2%, 6.4%, and 1.7% of total deaths in China for year 2017. *P* is the exposed population for each city in the YRD region and is obtained from statistical yearbooks for year 2018. The exposed PM_2.5_ concentration in Equation 1 is the PM_2.5_ concentrations averaged during each period. The threshold PM_2.5_ concentration *C*_*0*_ per World Health Organization (WHO) air quality guidelines (WHO, 2005) of 25 μg/m^3^ is used. We calculate the number of premature mortality using observed PM_2.5_ concentrations during pre‐lockdown, Level I, and Level II period of 2017–2020 to show trends over the past 4 years. Adjusted PM_2.5_ concentrations calculated based on the RF method (section 2.1) are used to calculate the number of premature mortality assuming no lockdown occurred. The differences between results obtained based on observed and adjusted PM_2.5_ concentrations illustrate the health impacts due to changes in air quality during lockdown periods.

## 3 Results and Discussions

### 3.1 Changes of PM_2.5_ Concentrations During COVID‐19 Lockdown

**Figure 2:**
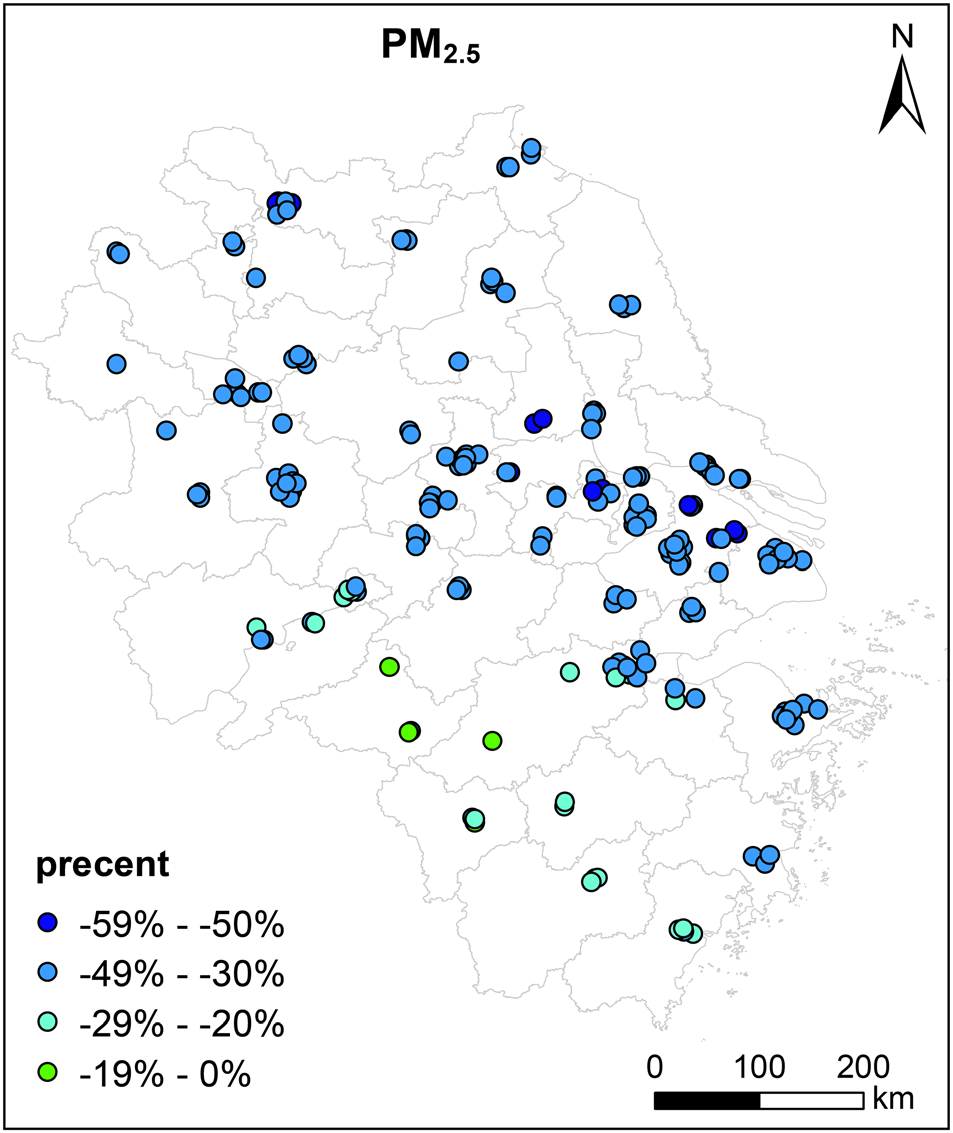
Relative changes of observed PM_2.5_ concentrations at 202 monitoring sites over the YRD region before (1st January to 23rd January 2020) and during lockdown (24th January to 31st March 2020).

Figure 2 shows the relative changes in observed PM_2.5_ concentrations before lockdown (1st January to 23rd January 2020) and lockdown period (24th January to 31st March 2020) for the YRD region. Averaged PM_2.5_ concentrations exhibit ubiquitous decreases across the YRD region during lockdown period with relative reductions by 22.9% (Huangshan in southern Anhui province) ~ 54.0% (Yangzhou in Jiangsu province). During pre‐lockdown period, Huaibei (in northern Anhui province) had the highest PM_2.5_ concentrations of 113.3 μg/m^3^ among all cities over the YRD region. The highest city‐level PM_2.5_ concentrations dropped to 64.9 μg/m^3^ in Bozhou (in northern Anhui province) during lockdown periods. Compared with same periods of previous years (averages of 2017–2019), PM_2.5_ concentrations during Level I response period decreased by 16.4% to 56.3% and by 4.8% to 50.9% during Level II response period (supporting information Tables S1–S4).

**Figure 3:**
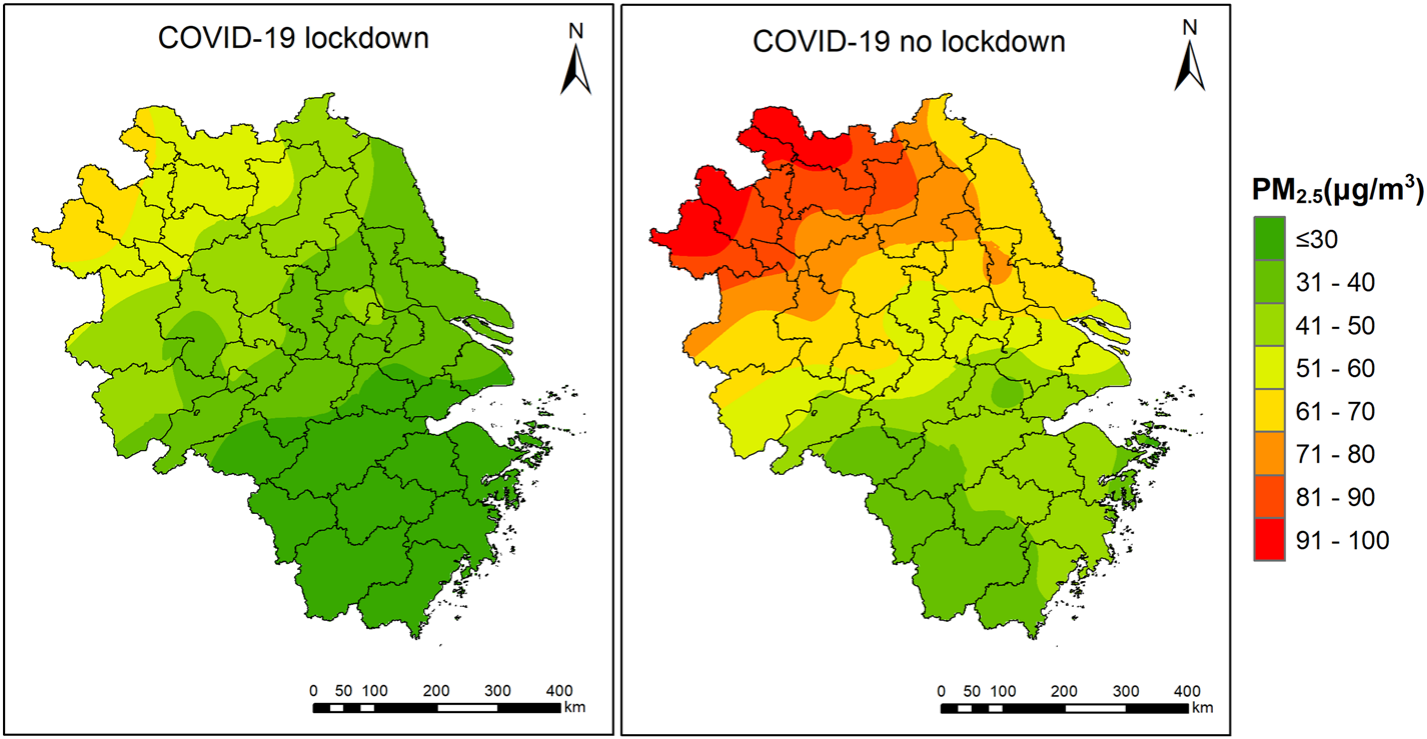
Spatial distribution of PM_2.5_ concentrations during lockdown period (left) and adjusted PM_2.5_ concentrations assuming no lockdown (right) over the YRD region.

Figure 3 compares the spatial distribution of PM_2.5_ concentrations with and without COVID‐19 lockdown. If no lockdown occurred (i.e., emissions were at baseline level), PM_2.5_ concentrations are estimated to be 27.1 (Zhoushan) to 97.5 μg/m^3^ (Bozhou), as compared to 18.7 (Zhoushan) to 64.9 μg/m^3^ (Bozhou). In Shanghai, Hangzhou, Nanjing, and Hefei, PM_2.5_ concentrations are estimated to be 52.4, 43.1, 53.5, and 70.8 μg/m^3^, which is 64.8%, 50.6%, 58.4%, and 85.1% higher than lockdown values.

### 3.2 Premature Mortality Attributable to Short‐Term PM_2.5_ Exposure

**Figure 4:**
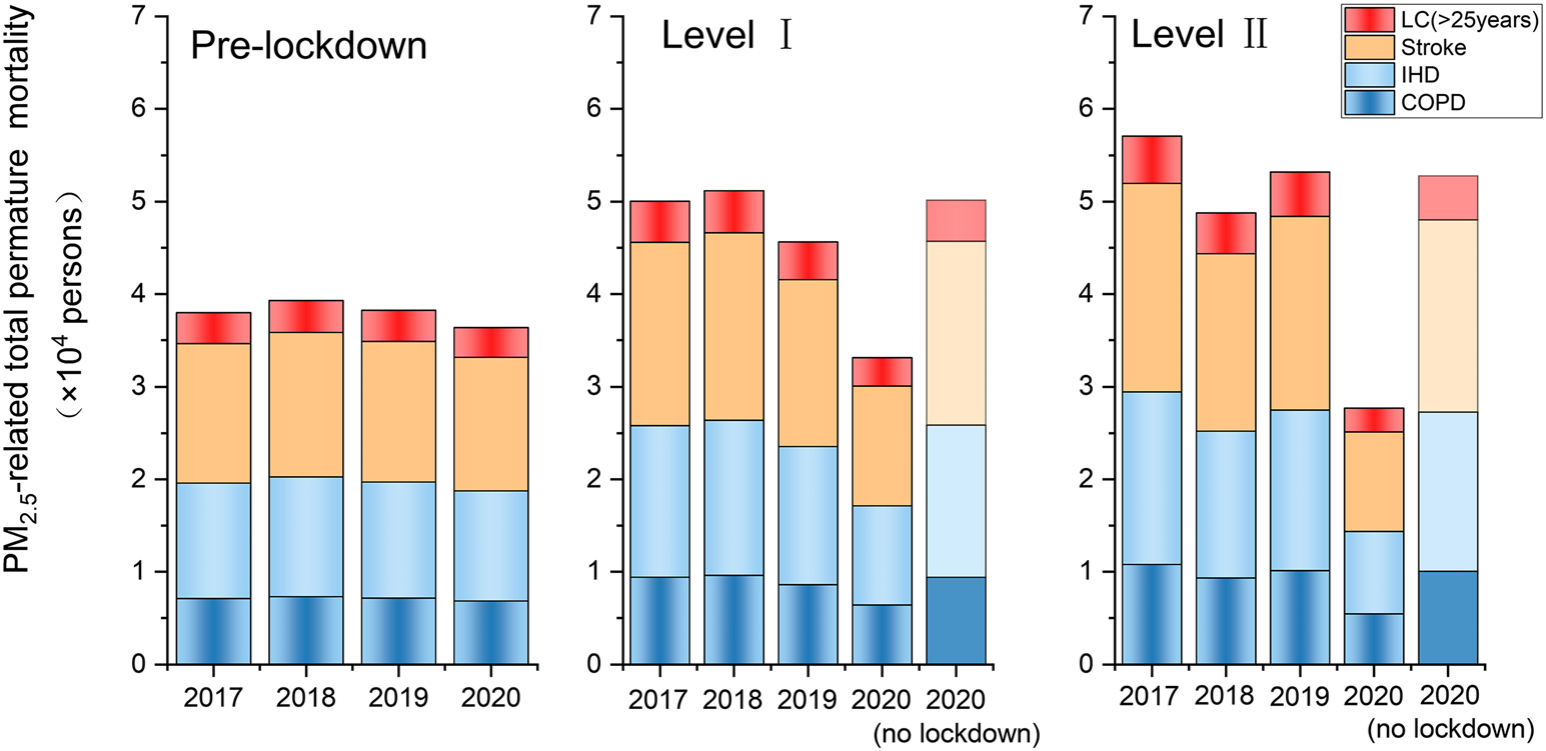
Premature mortality due to LC, stroke, IHD, COPD during pre‐lockdown, Level I, and Level II periods of 2017–2020 (data for ALRI are not shown due to low numbers). Estimated premature mortality with the assumption of no‐lockdown is also shown for Level I and Level II.

Ambient PM_2.5_ exposure leads to higher mortality in infants (<5 years) from ALRI and in adults (≥25 years) due to stroke, IHD, COPD, and LC. We calculate the premature mortality due to above‐mentioned causes based on the health impact function (Equation 1) over the YRD region during pre‐lockdown, Level I, and Level II period of 2017–2020 (Figure 4). During the pre‐lockdown period, the total premature mortality attributed to PM_2.5_ exposure are relatively consistent during 2017–2020 and the number in 2020 is 36.4 thousand (95% CI: 30.4–38.8 thousand) for the whole YRD region. Stroke and IHD contribute to 14.4 thousand (95% CI: 12.0–15.4 thousand) and 11.9 thousand (95% CI: 10.0–12.7 thousand) premature death, together accounting for 72.2% of total PM_2.5_‐related premature death. COPD, LC, and ALRI contribute the rest 27.8% of PM_2.5_‐related death, each causing 6.8 thousand (95% CI: 5.7–7.2 thousand), 3.2 thousand (95% CI: 2.7–3.4 thousand), and 0.05 thousand (95% CI: 0.04–0.06 thousand) premature death during the pre‐lockdown period. During Level I and Level II response periods, while the premature morality due to PM_2.5_ exposure during 2017–2019 fluctuated a little bit, a sharp decrease is observed for year 2020. The total PM_2.5_‐related premature mortality during 2020 Level I and Level II period is 33.2 thousand (95% CI: 23.9–38.5 thousand) and 27.7 thousand (95% CI: 18.6–33.8 thousand), which dropped by 32.3% and 47.7% compared to the 2017–2019 average values. The relative contributions from different diseases remain unchanged since the only changing variable here is the PM_2.5_ concentration.

In order to directly compare the premature mortality before and during lockdown (Level I plus Level II period), we present the daily premature mortality in Figure S1. During 2017–2019, the daily premature morality dropped by 3.2% to 12.7% before and during lockdown. For 2020, the daily premature mortality across the YRD region during pre‐lockdown is 1.5 thousand (95% CI: 1.3–1.6 thousand) and 0.9 thousand (95% CI: 0.6–1.1 thousand) during lockdown (Level I + Level II), representing a sharp decrease by 43.3%. The significant reduction in premature mortality during lockdown periods, whether it is compared to the pre‐lockdown periods of the same year, or the same periods of historical years, indicate substantial health benefits associated with lowered PM_2.5_ concentrations due to COVID‐19 lockdown.

**Figure 5:**
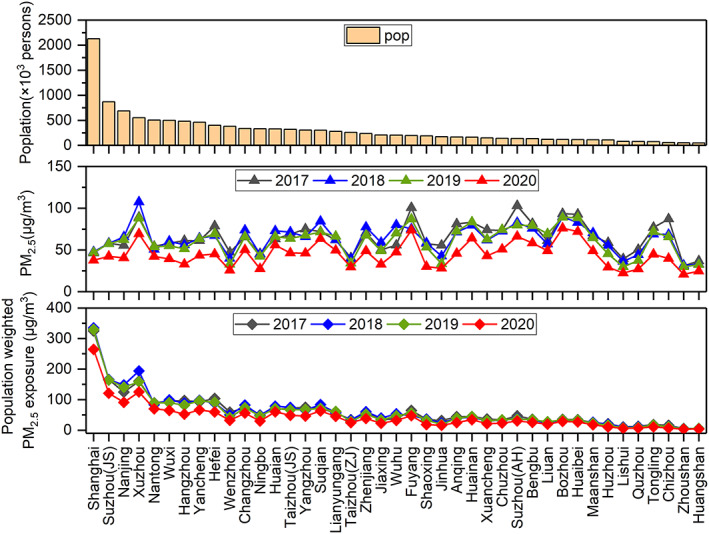
City‐level population, averaged PM_2.5_ concentration, and population weighted PM_2.5_ exposure (calculated as city‐level population multiplied with PM_2.5_ concentration divided by averaged population across all cities) during January to March of 2017–2019 for 41 cities over the YRD region.

Tables S1–S4 show the city‐level premature mortality by period and year. With the same base incidence rate (R) being applied for all cities, city‐level premature mortality depends on the exposed population and the PM_2.5_ concentration for that city. With the largest population (2418.3 thousand in 2018) of all cities in the YRD region (Figure 5), Shanghai has the highest PM_2.5_‐related premature mortality during 2017–2019, even though its averaged PM_2.5_ concentration is ranked the sixth, ninth, and ninth from the bottom during January to March of 2017, 2018, and 2019 (Figure 5). On the other hand, high premature mortality for cities like Bozhou and Suqian in Anhui province (e.g., each is ranked third and seventh during Level II period in 2020 in terms of premature mortality) is more associated with the high PM_2.5_ concentrations, which ranked third and seventh in terms of average PM_2.5_ concentration and only thirteenth and nineteenth in terms of population. Cities with small population and low PM_2.5_ concentrations, for example, Huangshan (in Anhui province), have the lowest premature mortality. During 2020 pre‐lockdown, daily premature mortality due to PM_2.5_ exposure in Shanghai is 145 (95% CI: 112–158). This number drops to 84 (95% CI: 53–106) during Level I period and 25 (95% CI: 14–35) during Level II period, each representing a decrease by 42.1% and 82.8% compared to pre‐lockdown level. During 2020 Level II period, the number of premature death due to PM_2.5_ exposure in Shanghai drops to the twelfth place due to a sharp decrease in PM_2.5_ concentrations during Level II (decrease by 24.0%). The number of cities with zero PM_2.5_‐related premature mortality (i.e., PM_2.5_ concentration below threshold value) increases from zero during 2020 pre‐lockdown period to five (Huangshan in Anhui province, Lishui, Quzhou, Wenzhou, and Zhoushan in Zhejiang province) during Level I and six (Huzhou, Jiaxing, Lishui, Ningbo, Wenzhou, and Zhoushan in Zhejiang province) during Level II period.

### 3.3 Avoided Premature Mortality Due to PM_2.5_ Improvement During COVID‐19 Lockdown

An integrated WRF/CAMx model is used to estimate PM_2.5_ concentrations that would be during Level I and Level II period if no lockdown occurred. The potential health benefits due to lockdown are estimated as the differences of premature mortality calculated based on the observed PM_2.5_ concentrations and the simulated “no lockdown” concentrations during the same periods. If no lockdown occurred, the total premature death due to PM_2.5_ exposure over the YRD region would be 50.2 thousand (95% CI: 42.0–53.3 thousand) during Level I period and 52.9 thousand (95% CI: 40.6–58.5 thousand) during Level II period. These two numbers are lower than the corresponding values of 2017–2019 (except for Level I in 2017 and 2019). The total avoided premature mortality over the YRD region is 17.1 thousand during Level I and 25.1 thousand during Level II (Table S5), each representing 51.5% and 90.6% of current premature mortality rate due to PM_2.5_ exposure. In terms of diseases, avoided premature morality due to IHD and stroke are 14.0 thousand and 16.9 thousand, each accounting for 33.2% and 40.1% of current base incident mortality rate due to PM_2.5_. Avoided premature mortality due to COPD, LC, and ALRI are 7.6 thousand, 3.6 thousand, and 0.06 thousand.

**Figure 6:**
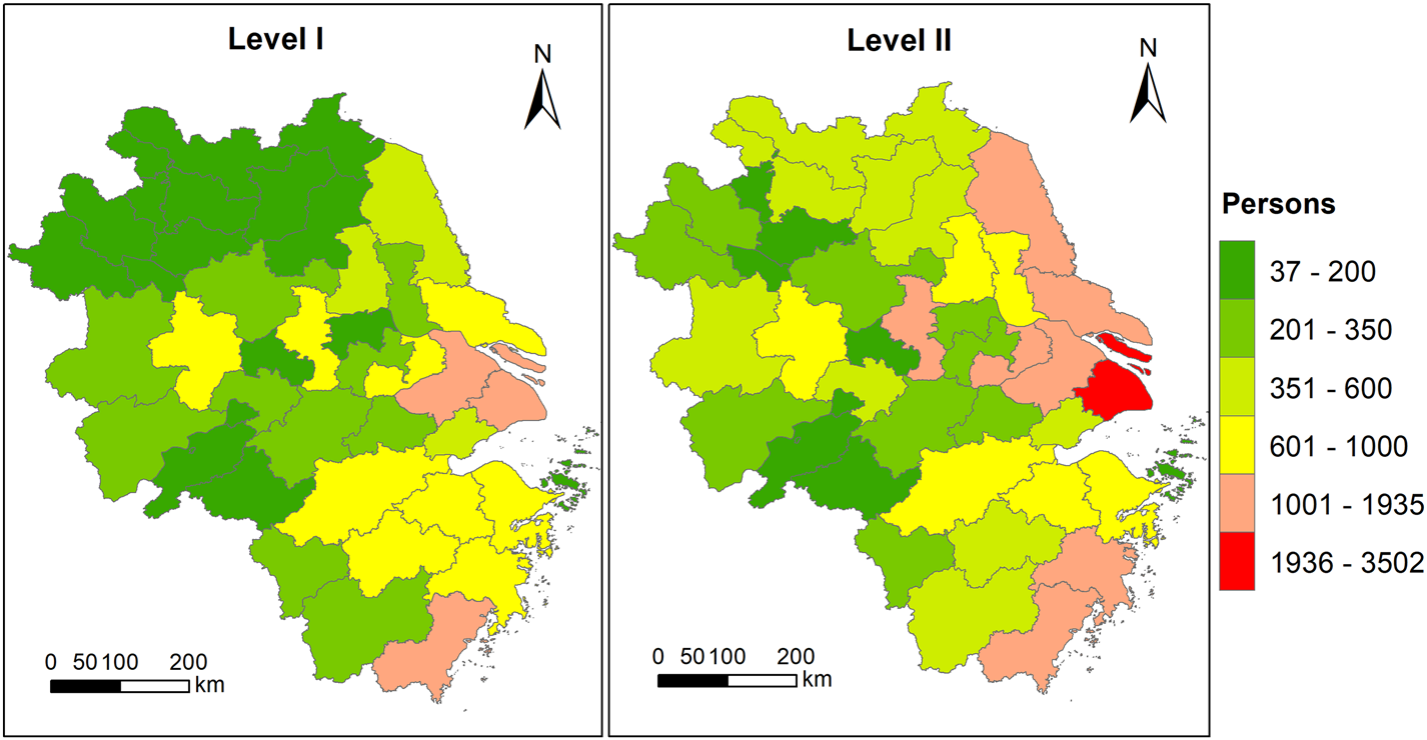
Spatial distribution of avoided total premature death during Level I and Level II.

Figure 6 shows the city‐level total avoided premature mortality during Level I and Level II (avoided premature mortality associated with different diseases are shown in Figure S2). The number of avoided premature death for different cities depends on the base population and the changes in PM_2.5_ concentrations due to lockdown (Table S5). During Level I period, the top five cities with largest avoided premature death are Shanghai (1,932), Wenzhou (1,124), Suzhou (Jiangsu province; 1,118), Ningbo (934), and Hangzhou (934). During Level II period, the top five cities with largest avoided premature death are Shanghai (3,502), Wenzhou (1,628), Suzhou (Jiangsu province; 1,542), Nantong (1,270), and Nanjing (1,227). It should be noted that the higher number of avoided premature death during Level II period over Level I period is simply because Level II covers more days (36 days) than Level I (31 days). The daily avoided deaths during Level I are higher than Level II due to more restricted transportation and industrial activities.

### 3.4 Premature Mortality Due to Residual PM_2.5_ During COVID‐19 Lockdown

Although the PM_2.5_ concentrations during COVID‐19 lockdown seem to have dropped substantially whether compared with pre‐lockdown periods or the same periods of previous years, there are still 36 (during Level I) and 35 (during Level II) out of 41 cities with residual PM_2.5_ concentrations that are higher than the recommended 24‐hr PM_2.5_ concentrations of 25 μg/m^3^ by WHO. Premature mortality caused by PM_2.5_‐exposure during Level I and Level II period is estimated to be 33.2 thousand (95% CI: 23.9–38.4 thousand) and 27.7 thousand (95% CI: 18.6–33.8 thousand), respectively, each representing 31.2% and 22.4% of total death rate over the YRD region. The top five cities with highest premature mortality during Level I are Shanghai (2,687, 95% CI: 1,707–3,385), Fuyang (2,581, 95% CI: 2,197–2,678), Xuzhou (2,229, 95% CI: 1,835–2,348), Suzhou (Jiangsu province, 1,606, 95% CI: 1,021–2,023), and Bozhou (1,599, 95% CI: 1,406–1,639). During Level II period, the top five cities with highest premature mortality are Fuyang (2,647, 95% CI: 1,996–2,929), Xuzhou (2,226, 95% CI: 1,627–2,514), Bozhou (1,587, 95% CI: 1,185–1767), Suzhou (Jiangsu province, 1,459, 95% CI: 890–1,906), and Suzhou (Anhui province, 1,449, 95% CI: 1,027–1,672).

### 3.5 Uncertainties

A few of uncertainties exist with our estimation of health impacts, which are also recognized in a couple of similar studies (Liu et al., 2016; Maji et al., 2018; Y. Wang et al., 2020). First and foremost, there exists uncertainties with the parameters (e.g., concentration‐response coefficient, threshold concentration) used in the integrated exposure‐response function. Although limitation exists, we use values reported by Lu, Xu, et al. (2015), which is developed based on meta‐analysis of 59 studies covering 22 cities in Mainland China (three cities in the YRD region), Hong Kong, and Taiwan to reduce uncertainties with the concentration‐response coefficient. Other simple assumptions when using the exposure‐response function include that the exposure‐response coefficient does not vary by age and a provincial‐level base incidence rate is used for all cities within the province. Second, we only calculate the premature mortality associated with PM_2.5_ exposure while realizing that synergistic effects exist when exposed to other or multiple pollutants (Apte et al., 2015; Billionnet et al., 2012) and our estimation of premature mortality perhaps represents an underestimation. On top of that, the influences of PM_2.5_ chemical composition, size distribution, and sources on health impacts (Ostro et al., 2015) are ignored in this study. When we calculate city‐level population exposure, we ignore the heterogeneities of ambient PM_2.5_ concentrations and population within the city. We simply use the observed PM_2.5_ concentrations averaged over all monitoring sites within the city as the exposed concentrations. This introduces underestimation when more monitoring sites are located in the rural and less populated areas and overestimation when monitoring sites are more likely to be located in urban and populous areas for a city. Ways to improve the spatial accuracy of health impact estimation include utilizing population distribution with high spatial resolution and interpolated PM_2.5_ concentrations based on networks of low‐cost sensors (Cavaliere et al., 2018; J. Li et al., 2020; Holstius et al., 2014) or satellite based data (e.g., the aerosol optical depth, Chen et al., 2019; J. Li et al., 2020). Finally, estimations of avoided premature death assuming no‐lockdown are associated with uncertainties with the meteorology and air quality model. This may all be explored in the near‐future when more data are available.

## 4 Conclusions

In this study, we attempt to quantify the health impacts associated with PM_2.5_ improvement due to reduced human and industrial activities during COVID‐19 lockdown in the YRD region, a region with heavy air pollution during the past years. As a result of reduced human activities, concentrations of PM_2.5_ during lockdown periods reduced by 22.9% to 54.0% over the YRD region compared to pre‐lockdown level. Avoided premature death due to lowered PM_2.5_ concentrations are estimated to be 42.2 thousand over the YRD region, representing 69.3% of the total present premature mortality due to PM_2.5_ exposure. The avoided premature mortality is mostly contributed by reduced death associated with stroke (16.9 thousand, accounting for 40.0%), ischemic heart disease (14.0 thousand, 33.2%), and chronic obstructive pulmonary disease (7.6 thousand, 18.0%). Top five cities with highest avoided premature mortality are Shanghai (5,433 persons), Wenzhou (2,751), Suzhou (in Jiangsu province, 2,660), Nanjing (2,000), and Nantong (1,969).

The outbreak of COVID‐19 is disastrous. This study is by no means to suggest that pandemics are bringing a positive effect for health. The passive emission reductions during COVID‐19 lockdown provide a good opportunity to show the health impacts related to reduction in PM_2.5_. It merely reinforces our knowledge that PM_2.5_ has detrimental health effects. Results from our study suggested that substantial health benefits could be achieved with reduced PM_2.5_ concentrations by emission reductions, which confirms the importance of recent efforts to mitigate the haze pollution nationwide. However, although PM_2.5_ concentration decreased substantially during lockdown period, the residual PM_2.5_ concentrations are still much higher than the recommended 24‐hr standard by WHO. Continuous efforts are needed to reduce emissions in the long term and through the most cost‐effective ways in order to protect public health.

## Conflict of Interest

The authors declare no conflict of interest relevant to this study.

## Supporting information

Supporting Information S1
