# Supplementary material for: The Silver Lining of COVID‐19: Estimation of Short‐Term Health Impacts Due to Lockdown in the Yangtze River Delta Region, China
Source: Geohealth. 2020 Sep 1;4(9):e2020GH000272. doi: 10.1029/2020GH000272 (PMC7361223; doi:10.1029/2020GH000272)
Supplement: Supplementary file 1 — Supporting Information S1 [file GH2-4-e2020GH000272-s001.docx]

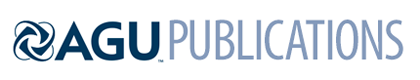


*GeoHealth*

Supporting Information for

**The silver lining of COVID-19: estimation of short-term health impacts due to lockdown in the Yangtze River Delta region, China**

Ling Huang^1,2^, Ziyi Liu^1,2^, Hongli Li^1,2^, Yangjun Wang^1,2^, Yumin Li^3^, Yonghui Zhu^1,2^, Maggie Chel Gee Ooi^4,5^, Jing An^1,2^, Yu Shang^1,2^, Dongping Zhang^1,2^, Andy Chan^4^, Li Li^1,2*^

^1^School of Environmental and Chemical Engineering, Shanghai University, Shanghai, 200444, China.

^2^Key Laboratory of Organic Compound Pollution Control Engineering (MOE), Shanghai University, Shanghai 200444, China.

^3^SILC Business School, Shanghai University, Shanghai, 2000444, China.

^4^Department of Civil Engineering, University of Nottingham Malaysia, Semenyih 43500, Selangor, Malaysia.

^5^Institute of Climate Change (IPI), National University of Malaysia (UKM), 43600, Bangi, Selangor, Malaysia


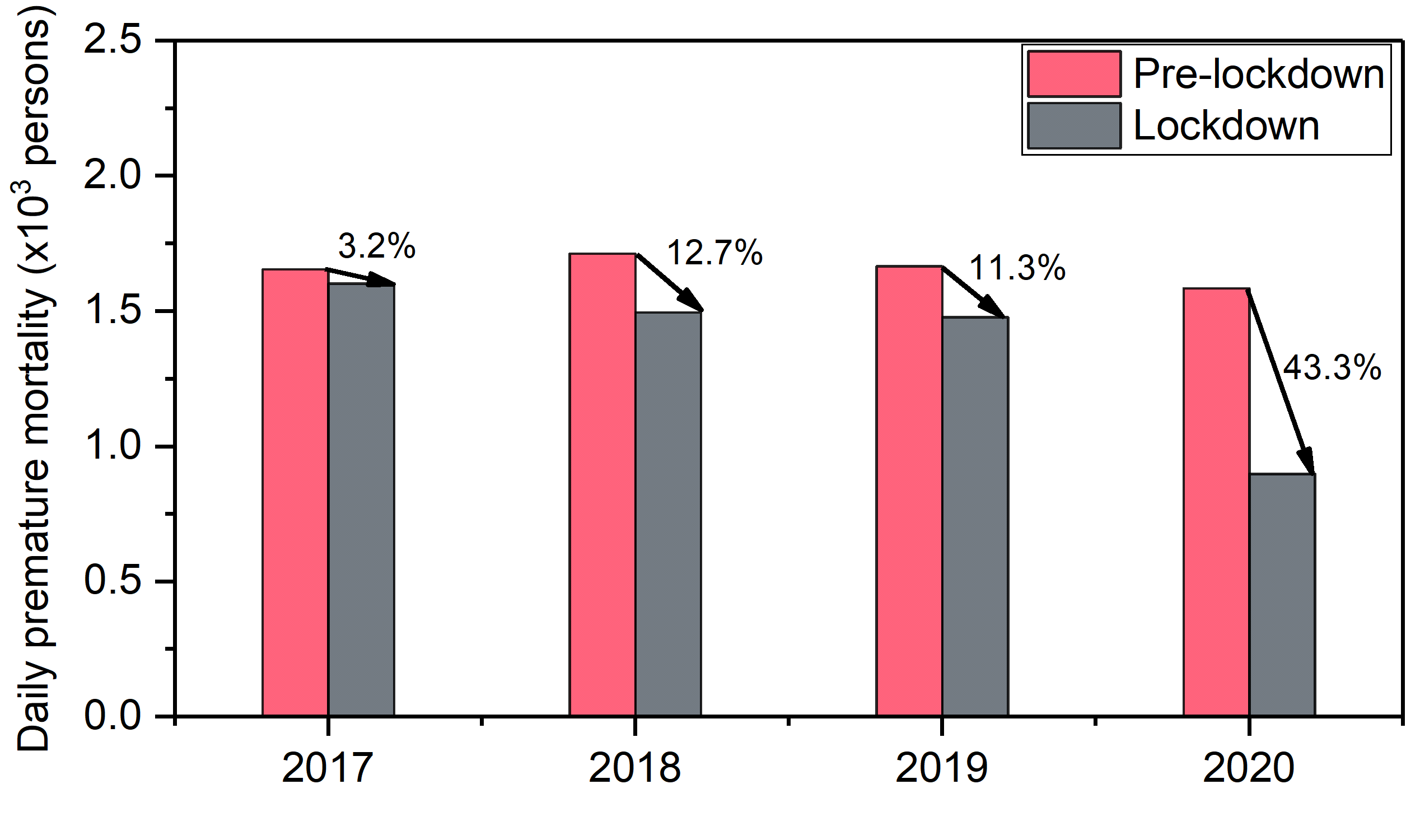


**Figure S1.** Estimated daily premature mortality during pre-lockdown and lockdown periods of 2017-2020


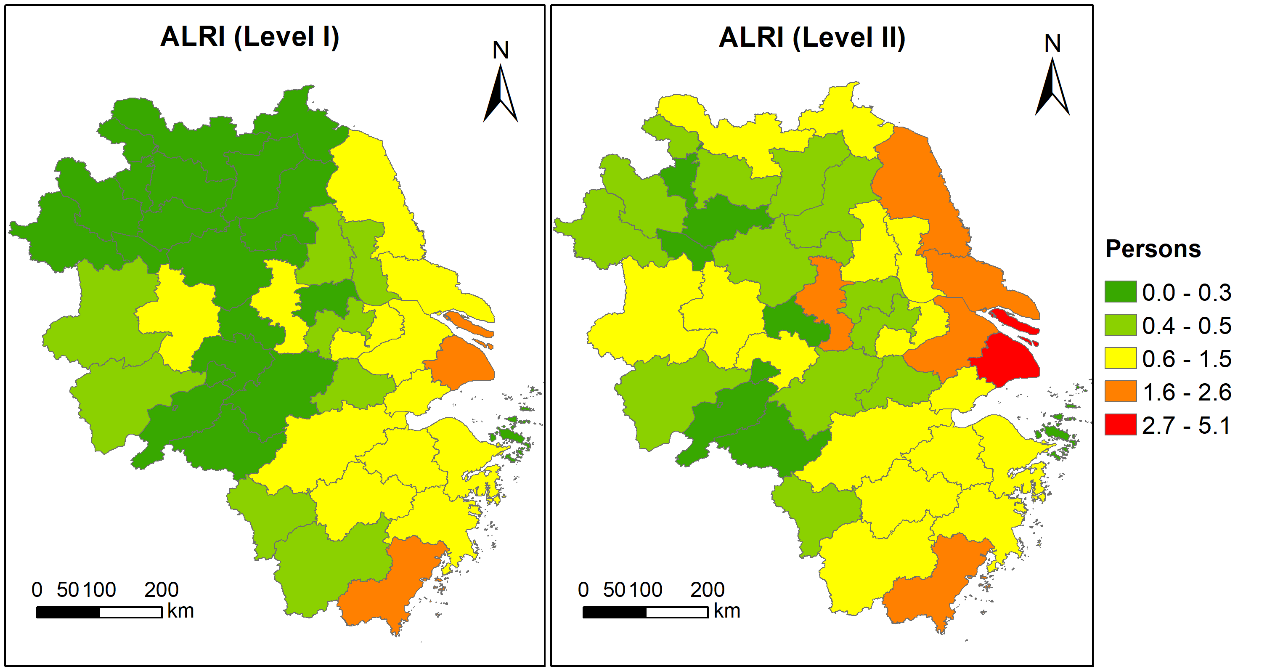


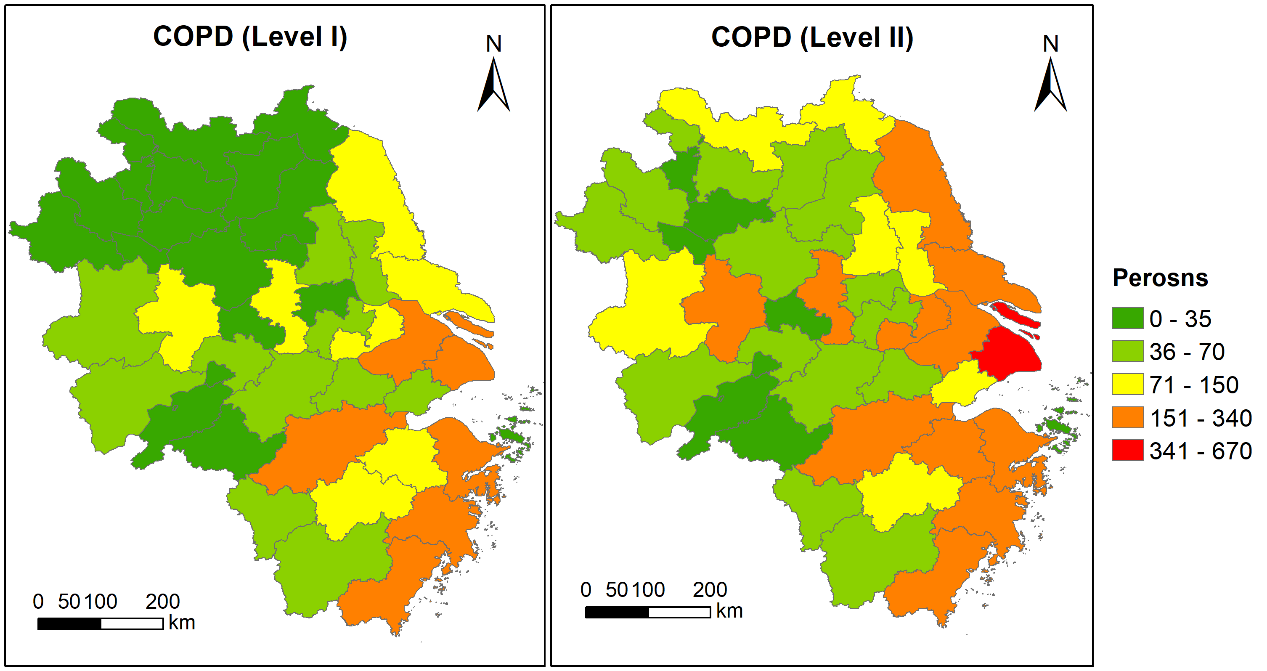


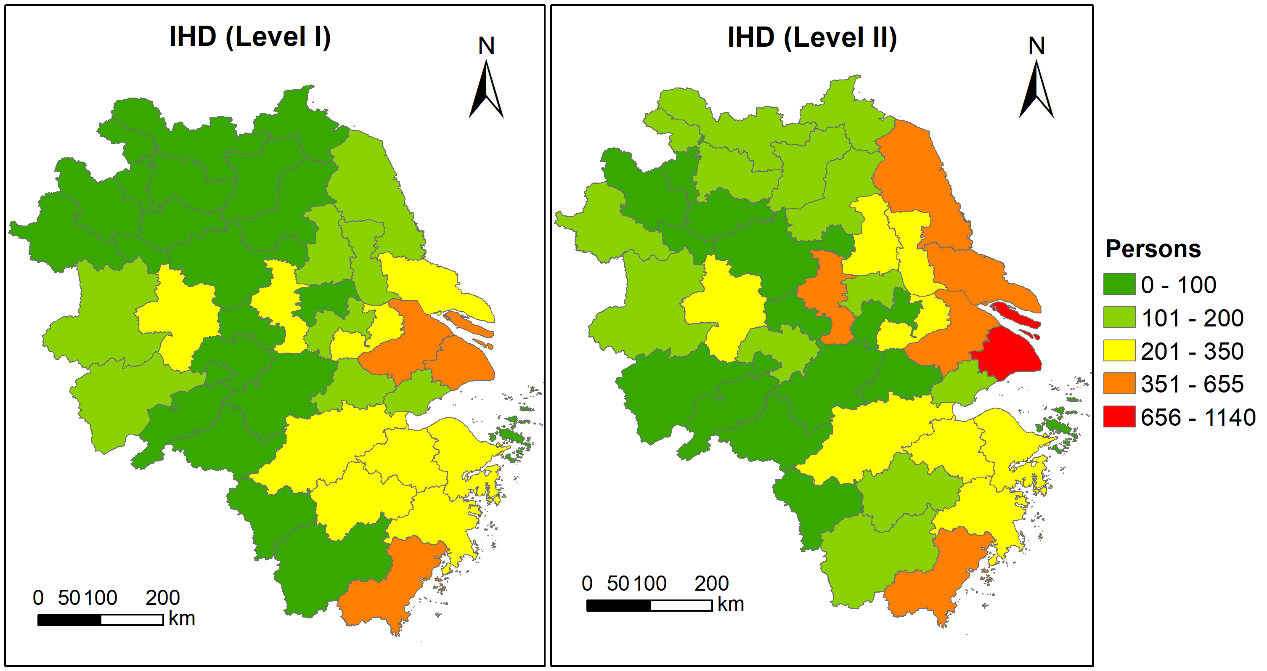


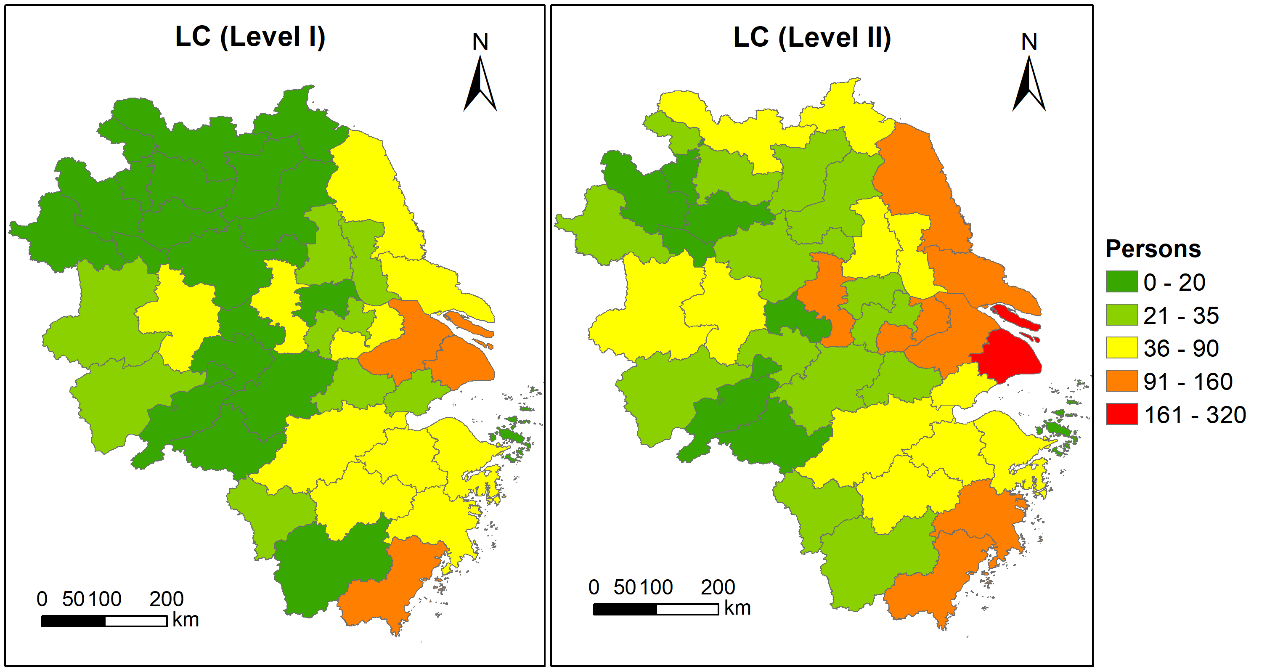


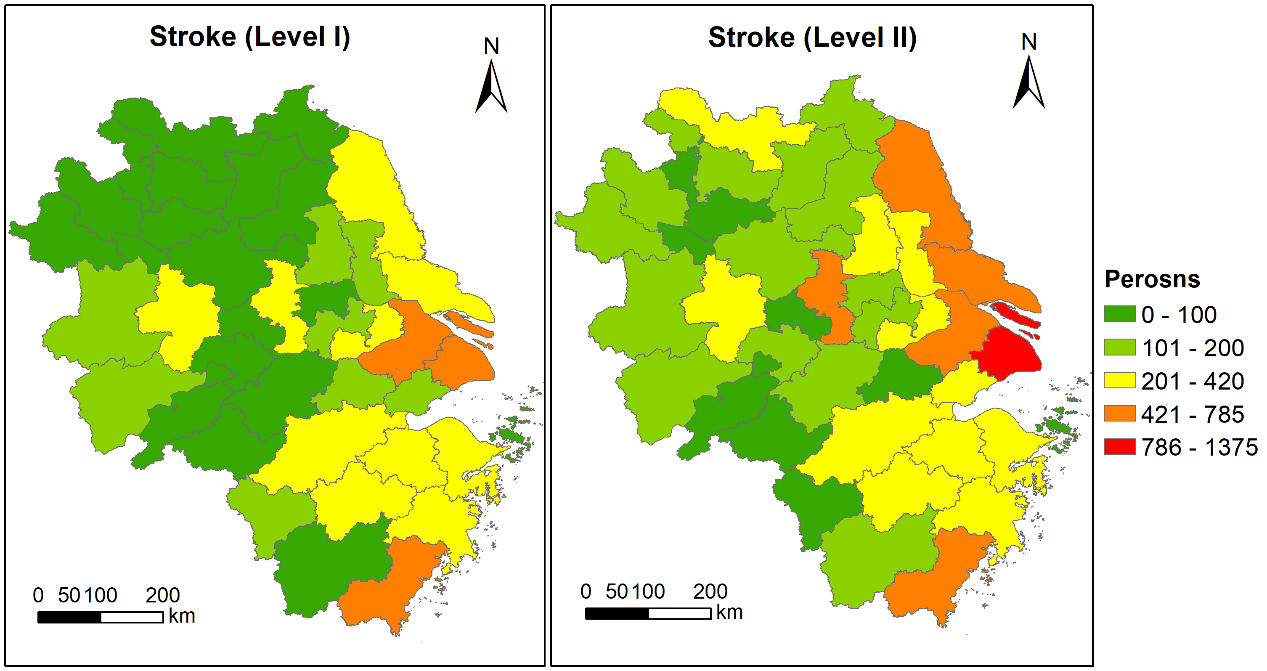


**Figure S2.** City-level avoided premature mortality due to ALRI, COPD, IHD, LC, and Stroke during Level I and Level II

| Province | City | Pre-lockdown | | Level Ⅰ | | Level Ⅱ | |
| --- | --- | --- | --- | --- | --- | --- | --- |
|  |  | PM_2.5_ | premature mortality | PM_2.5_ | premature mortality | PM_2.5_ | premature mortality |
|  |  |  |  |  |  |  |  |
| Shanghai | | 47.5 | 2,952 (2,122-3,374) | 44.3 | 3,702 (2,576-4,332) | 47.6 | 4,628 (3,328-5,286) |
| Zhejiang | Hangzhou | 67.3 | 1,227 (1,023-1,285) | 57.1 | 1,551 (1,209-1,681) | 61.0 | 1,856 (1,488-1,981) |
|  | Huzhou | 63.2 | 425 (386-458) | 56.9 | 546 (475-609) | 57.4 | 637 (467-677) |
|  | Jiaxing | 48.6 | 488 (354-554) | 49.0 | 663 (483-750) | 52.3 | 808 (606-897) |
|  | Jinhua | 62.3 | 726 (587-772) | 54.1 | 914 (696-1,005) | 53.1 | 1,048 (791-1,158) |
|  | Lishui | 38.4 | 275 (376-461) | 39.7 | 391 (446-605) | 39.5 | 451 (516-702) |
|  | Ningbo | 47.7 | 805 (580-918) | 41.7 | 934 (633-1,116) | 47.7 | 1,260 (907-1,437) |
|  | Quzhou | 58.4 | 398 (313-429) | 50.7 | 492 (364-551) | 44.9 | 512 (358-596) |
|  | Shaoxing | 64.6 | 717 (589-757) | 52.6 | 875 (658-969) | 57.5 | 1,070 (837-1,158) |
|  | Taizhou | 38.1 | 614 (1,035-1,062) | 42.8 | 990 (1,340-1,430) | 39.9 | 1,040 (1,490-1,658) |
|  | Wenzhou | 41.4 | 945 (538-1,023) | 43.5 | 1,361 (914-1,579) | 54.8 | 1,924 (930-1,701) |
|  | Zhoushan | 29.7 | 45 (27-62) | 31.4 | 78 (47-104) | 31.9 | 97 (58-128) |
| Jiangsu | Changzhou | 72.1 | 892 (763-924) | 66.5 | 1,177 (977-1,235) | 59.7 | 1,313 (1,044-1,409) |
|  | Huaian | 75.0 | 986 (989-1,025) | 64.9 | 1,281 (1,249-1,378) | 53.8 | 1,364 (1,365-1,593) |
|  | Lianyungang | 70.3 | 849 (344-648) | 59.1 | 1,078 (495-910) | 58.9 | 1,250 (570-1,051) |
|  | Nanjing | 63.3 | 1,517 (1,235-1,607) | 55.2 | 1,919 (1,474-2,099) | 50.3 | 2,095 (1,544-2,352) |
|  | Nantong | 57.5 | 1,336 (1,045-1,446) | 56.9 | 1,791 (1,394-1,943) | 49.9 | 1,912 (1,405-2,151) |
|  | Suzhou | 61.7 | 2,073 (2,062-2,272) | 57.3 | 2,703 (2,600-3,047) | 56.3 | 3,111 (2,343-3,355) |
|  | Taizhou | 72.9 | 913 (699-925) | 72.1 | 1,228 (885-1,227) | 63.4 | 1,373 (1,010-1,418) |
|  | Wuxi | 65.1 | 1,205 (1,161-1,294) | 55.2 | 1,510 (1,496-1,738) | 56.2 | 1,771 (1,608-1,999) |
|  | Suqian | 90.6 | 991 (645-938) | 77.2 | 1,310 (904-1,281) | 54.7 | 1,351 (1,079-1,499) |
|  | Xuzhou | 118.4 | 1,777 (1,561-1,774) | 86.7 | 2,358 (1,938-2,369) | 71.9 | 2,659 (2,261-2,753) |
|  | Yancheng | 63.9 | 1,340 (1,095-1,417) | 63.8 | 1,806 (1,474-1,909) | 56.7 | 1,993 (1,549-2,164) |
|  | Yangzhou | 80.5 | 887 (788-906) | 77.4 | 1,188 (1,042-1,219) | 69.2 | 1,348 (1,136-1,405) |
|  | Zhenjiang | 70.9 | 632 (538-657) | 73.9 | 860 (742-888) | 67.9 | 979 (819-1,023) |
| Anhui | Anqing | 98.8 | 1,008 (944-1,015) | 86.4 | 1,345 (1,219-1,365) | 64.9 | 1,475 (1,212-1,554) |
|  | Bengbu | 94.7 | 710 (659-716) | 84.8 | 947 (854-963) | 70.4 | 1,065 (903-1,107) |
|  | Bozhou | 116.4 | 1,221 (1,178-1,224) | 103.2 | 1,640 (1,551-1,649) | 69.9 | 1,815 (1,535-1,889) |
|  | Chizhou | 105.6 | 304 (289-305) | 91.4 | 406 (374-411) | 71.6 | 456 (389-472) |
|  | Chuzhou | 80.7 | 832 (739-850) | 77.5 | 1,115 (977-1,144) | 64.8 | 1,240 (1,019-1,307) |
|  | Fuyang | 121.7 | 2,009 (1,950-2,012) | 105.9 | 2,699 (2,565-2,711) | 81.9 | 3,075 (2,744-3,136) |
|  | Hefei | 89.0 | 1,469 (1,342-1,488) | 83.7 | 1,967 (1,767-2,002) | 67.0 | 2,186 (1,820-2,291) |
|  | Huaibei | 118.5 | 407 (382-408) | 90.2 | 542 (482-548) | 77.9 | 619 (518-631) |
|  | Huainan | 101.7 | 728 (374-622) | 83.3 | 966 (468-809) | 71.3 | 1,092 (53-136) |
|  | Huangshan | 44.8 | 204 (209-272) | 42.8 | 260 (257-358) | 26.3 | 36 (301-417) |
|  | Luan | 67.7 | 1,039 (869-1,087) | 65.0 | 1,384 (1,138-1,458) | 53.4 | 1,466 (1,110-1,617) |
|  | Ma'anshan | 69.5 | 408 (344-425) | 67.5 | 546 (455-571) | 64.7 | 626 (514-660) |
|  | Tongling | 87.5 | 316 (265-318) | 78.7 | 421 (354-429) | 68.6 | 475 (377-491) |
|  | Wuhu | 53.5 | 617 (325-577) | 55.6 | 851 (479-816) | 57.2 | 1,004 (751-1,074) |
|  | Suzhou | 127.5 | 1,231 (942-1,205) | 101.7 | 1,651 (1,102-1,568) | 88.1 | 1,899 (1,302-1,831) |
|  | Xuancheng | 83.1 | 516 (509-527) | 70.7 | 676 (634-709) | 71.3 | 786 (673-816) |
| Total |  |  | 38,036 (32,175-40,039) |  | 50,122 (41,187-53,485) |  | 57,165 (44,730-60,980) |

**Table S1**. Averaged PM_2.5_ concentrations (μg/m^3^) and PM_2.5_-related premature morality by city during pre-lockdown, level I, and level II periods in 2017

| Province | City | Pre-lockdown | | Level Ⅰ | | Level Ⅱ | |
| --- | --- | --- | --- | --- | --- | --- | --- |
|  |  | PM_2.5_ | premature mortality | PM_2.5_ | premature mortality | PM_2.5_ | premature mortality |
|  |  |  |  |  |  |  |  |
| Shanghai | | 54.5 | 3,270 (2,498-3,589) | 51.2 | 4,232 (3,145-4,725) | 40.0 | 3,765 (2,503-4,564) |
| Zhejiang | Hangzhou | 65.2 | 1,215 (1,001-1,280) | 62.4 | 1,613 (1,305-1,713) | 43.9 | 1,459 (1,012-1,712) |
|  | Huzhou | 65.8 | 430 (431-462) | 61.0 | 564 (477-609) | 42.5 | 496 (534-702) |
|  | Jiaxing | 66.7 | 579 (481-607) | 67.4 | 782 (653-819) | 45.9 | 725 (513-837) |
|  | Jinhua | 49.1 | 633 (462-715) | 47.5 | 828 (595-947) | 33.5 | 535 (329-695) |
|  | Lishui | 44.6 | 339 (404-465) | 42.6 | 432 (455-609) | 25.8 | 39 (460-678) |
|  | Ningbo | 51.4 | 854 (635-952) | 52.9 | 1,173 (885-1,299) | 32.8 | 656 (400-858) |
|  | Quzhou | 54.8 | 385 (295-421) | 48.7 | 476 (346-540) | 33.0 | 287 (175-375) |
|  | Shaoxing | 70.3 | 735 (623-764) | 64.2 | 965 (790-1,019) | 45.0 | 888 (623-1,034) |
|  | Taizhou | 40.5 | 680 (1,023-1,062) | 48.3 | 1,124 (1,198-1,419) | 30.7 | 522 (1,314-1,635) |
|  | Wenzhou | 48.3 | 1,122 (612-1,107) | 40.3 | 1,227 (1,097-1,721) | 34.4 | 1,041 (416-945) |
|  | Zhoushan | 32.3 | 64 (39-85) | 37.8 | 130 (84-161) | 25.5 | 8 (5-12) |
| Jiangsu | Changzhou | 91.6 | 922 (849-933) | 74.8 | 1,211 (1,050-1,248) | 60.6 | 1,322 (1,058-1,413) |
|  | Huaian | 99.9 | 1,017 (992-1,025) | 65.4 | 1,284 (1,160-1,370) | 62.2 | 1,465 (1,245-1,573) |
|  | Lianyungang | 80.6 | 871 (453-758) | 60.6 | 1,090 (566-982) | 52.5 | 1,172 (41-106) |
|  | Nanjing | 94.5 | 1,633 (1,515-1,648) | 65.3 | 2,067 (1,703-2,176) | 47.7 | 2,004 (1,442-2,287) |
|  | Nantong | 63.0 | 1,389 (1,128-1,473) | 57.9 | 1,807 (1,418-1,952) | 34.8 | 1,131 (706-1,446) |
|  | Suzhou | 73.1 | 2,180 (2,211-2,277) | 64.9 | 2,847 (2,517-3,035) | 41.3 | 2,347 (2,886-3,519) |
|  | Taizhou | 87.8 | 938 (785-944) | 70.3 | 1,220 (981-1,257) | 61.7 | 1,359 (663-1,179) |
|  | Wuxi | 78.0 | 1,258 (1,157-1,294) | 64.6 | 1,619 (1,469-1,735) | 45.0 | 1,490 (1,227-1,857) |
|  | Suqian | 123.7 | 1,003 (959-1,005) | 72.7 | 1,295 (1,205-1,350) | 71.1 | 1,497 (1,014-1,471) |
|  | Xuzhou | 147.3 | 1,781 (1,523-1,770) | 95.3 | 2,377 (1,879-2,358) | 94.5 | 2,758 (1,457-2,396) |
|  | Yancheng | 81.1 | 1,416 (1,260-1,446) | 59.1 | 1,751 (1,386-1,882) | 57.0 | 1,997 (1,556-2,166) |
|  | Yangzhou | 73.5 | 873 (752-902) | 65.2 | 1,140 (939-1,201) | 62.0 | 1,301 (1,050-1,384) |
|  | Zhenjiang | 100.6 | 660 (621-665) | 72.1 | 856 (732-886) | 66.7 | 974 (809-1,021) |
| Anhui | Anqing | 92.1 | 1,003 (925-1,014) | 79.2 | 1,330 (1,175-1,361) | 51.0 | 1,303 (966-1,457) |
|  | Bengbu | 103.3 | 713 (674-717) | 71.8 | 922 (787-955) | 62.6 | 1,028 (833-1,091) |
|  | Bozhou | 119.7 | 1,221 (1,183-1,224) | 88.1 | 1,623 (1,479-1,645) | 72.6 | 1,831 (1,570-1,895) |
|  | Chizhou | 89.8 | 301 (276-305) | 79.2 | 400 (353-409) | 46.0 | 358 (253-414) |
|  | Chuzhou | 100.4 | 848 (798-854) | 67.7 | 1,082 (904-1,132) | 59.1 | 1,196 (946-1,285) |
|  | Fuyang | 109.6 | 2,005 (1,916-2,012) | 72.4 | 2,592 (2,221-2,682) | 56.4 | 2,753 (2,136-2,993) |
|  | Hefei | 92.2 | 1,473 (1,359-1,489) | 69.0 | 1,899 (1,598-1,979) | 49.5 | 1,869 (1,368-2,109) |
|  | Huaibei | 120.6 | 407 (389-408) | 75.9 | 530 (472-547) | 66.6 | 597 (464-618) |
|  | Huainan | 110.3 | 730 (209-434) | 79.3 | 959 (470-810) | 60.9 | 1,039 (0-0) |
|  | Huangshan | 34.3 | 129 (215-273) | 42.9 | 261 (274-364) | 23.1 | 0 (205-356) |
|  | Luan | 74.8 | 1,064 (922-1,096) | 58.2 | 1,323 (1,040-1,428) | 45.3 | 1,276 (897-1,482) |
|  | Ma'anshan | 94.5 | 427 (396-431) | 75.9 | 561 (488-577) | 49.9 | 544 (400-613) |
|  | Tongling | 86.7 | 316 (288-321) | 75.9 | 418 (349-428) | 50.7 | 411 (369-488) |
|  | Wuhu | 111.0 | 726 (414-650) | 86.2 | 965 (417-756) | 53.7 | 969 (330-681) |
|  | Suzhou | 116.7 | 1,230 (1,050-1,225) | 75.4 | 1,601 (1,262-1,623) | 68.4 | 1,818 (993-1,646) |
|  | Xuancheng | 78.6 | 512 (521-527) | 67.4 | 667 (654-710) | 45.5 | 612 (758-824) |
| Total |  |  | 39,354 (34,244-40,629) |  | 51,243 (41,978-54,419) |  | 48,842 (35,926-53,817) |

**Table S2**. Averaged PM_2.5_ concentrations (μg/m^3^) and PM_2.5_-related premature morality by city during pre-lockdown, level I, and level II periods in 2018

| Province | City | Pre-lockdown | | Level Ⅰ | | | Level Ⅱ | |
| --- | --- | --- | --- | --- | --- | --- | --- | --- |
|  |  | PM_2.5_ | premature mortality | PM_2.5_ | | premature mortality | PM_2.5_ | premature mortality |
|  |  |  |  |  |  |  |  |  |
| Shanghai | | 53.4 | 3,231 (2,447-3,565) | 39.3 | | 3,151 (2,078-3,845) | 49.7 | 4,807 (3,527-5,415) |
| Zhejiang | Hangzhou | 63.1 | 1,202 (977-1,274) | 49.3 | 1,407 (1,028-1,590) | | 46.9 | 1,565 (1,118-1,795) |
|  | Huzhou | 58.2 | 410 (415-461) | 43.5 | 439 (303-517) | | 38.8 | 432 (432-658) |
|  | Jiaxing | 57.2 | 544 (425-590) | 46.8 | 636 (454-730) | | 45.5 | 717 (505-831) |
|  | Jinhua | 36.8 | 430 (275-539) | 31.4 | 372 (222-494) | | 33.0 | 513 (313-670) |
|  | Lishui | 35.8 | 239 (431-467) | 24.0 | - | | 31.1 | 242 (390-635) |
|  | Ningbo | 53.7 | 879 (667-969) | 38.5 | 823 (538-1,013) | | 38.4 | 953 (622-1,174) |
|  | Quzhou | 45.4 | 331 (233-384) | 34.2 | 275 (171-354) | | 35.2 | 343 (215-437) |
|  | Shaoxing | 66.4 | 724 (601-760) | 52.6 | 874 (658-969) | | 45.4 | 897 (632-1,041) |
|  | Taizhou | 39.6 | 656 (988-1,061) | 32.2 | 540 (326-713) | | 32.6 | 656 (1,235-1,616) |
|  | Wenzhou | 36.2 | 747 (585-1,077) | 26.0 | 127 (70-182) | | 36.9 | 1,216 (536-1,160) |
|  | Zhoushan | 33.8 | 74 (46-96) | 27.5 | 35 (20-49) | | 29.2 | 64 (37-88) |
| Jiangsu | Changzhou | 75.4 | 900 (782-926) | 61.1 | 1,142 (916-1,218) | | 64.4 | 1,353 (1,109-1,428) |
|  | Huaian | 88.7 | 1,009 (987-1,025) | 67.2 | 1,295 (1,079-1,356) | | 50.2 | 1,300 (1,231-1,570) |
|  | Lianyungang | 96.2 | 886 (289-578) | 68.2 | 1,135 (952-1,186) | | 46.1 | 1,053 (275-619) |
|  | Nanjing | 77.0 | 1,596 (1,397-1,639) | 59.8 | 2,000 (1,591-2,144) | | 54.9 | 2,222 (1,703-2,434) |
|  | Nantong | 61.7 | 1,378 (1,110-1,468) | 49.2 | 1,627 (1,188-1,839) | | 52.5 | 1,983 (1,491-2,198) |
|  | Suzhou | 64.4 | 2,106 (2,052-2,272) | 53.8 | 2,609 (1,982-2,872) | | 56.4 | 3,114 (2,410-3,383) |
|  | Taizhou | 74.9 | 918 (723-931) | 60.7 | 1,163 (931-1,243) | | 59.1 | 1,335 (1,012-1,419) |
|  | Wuxi | 63.7 | 1,196 (1,130-1,292) | 53.0 | 1,472 (1,111-1,628) | | 51.0 | 1,664 (1,549-1,986) |
|  | Suqian | 89.4 | 990 (897-1,002) | 76.0 | 1,307 (1,139-1,344) | | 56.3 | 1,372 (1,115-1,513) |
|  | Xuzhou | 108.7 | 1,774 (1,460-1,762) | 94.0 | 2,374 (2,200-2,397) | | 68.8 | 2,631 (1,885-2,647) |
|  | Yancheng | 76.9 | 1,405 (1,229-1,442) | 64.2 | 1,809 (1,481-1,911) | | 53.8 | 1,934 (1,470-2,129) |
|  | Yangzhou | 82.8 | 890 (797-907) | 62.8 | 1,125 (913-1,194) | | 61.6 | 1,297 (1,044-1,382) |
|  | Zhenjiang | 80.2 | 647 (574-661) | 64.3 | 829 (679-875) | | 62.4 | 952 (771-1,011) |
| Anhui | Anqing | 91.0 | 1,002 (921-1,014) | 76.1 | 1,321 (1,152-1,358) | | 56.4 | 1,389 (1,077-1,510) |
|  | Bengbu | 101.9 | 712 (672-717) | 77.6 | 936 (821-960) | | 65.4 | 1,043 (860-1,098) |
|  | Bozhou | 112.4 | 1,220 (1,171-1,224) | 104.0 | 1,640 (1,553-1,649) | | 59.9 | 1,723 (1,372-1,847) |
|  | Chizhou | 79.1 | 297 (262-304) | 72.0 | 393 (336-407) | | 50.8 | 391 (290-437) |
|  | Chuzhou | 88.4 | 841 (767-852) | 73.0 | 1,102 (947-1,139) | | 66.1 | 1,248 (1,033-1,310) |
|  | Fuyang | 109.6 | 2,005 (1,917-2,012) | 93.4 | 2,682 (2,481-2,708) | | 66.6 | 2,945 (2,445-3,089) |
|  | Hefei | 88.5 | 1,468 (1,340-1,488) | 68.1 | 1,891 (1,584-1,976) | | 57.7 | 2,062 (1,615-2,230) |
|  | Huaibei | 116.5 | 407 (384-408) | 93.7 | 544 (504-549) | | 65.5 | 594 (515-630) |
|  | Huainan | 103.6 | 728 (322-576) | 82.9 | 965 (865-983) | | 70.7 | 1,090 (205-469) |
|  | Huangshan | 41.1 | 183 (195-267) | 28.5 | 78 (45-108) | | 30.5 | 133 (171-320) |
|  | Luan | 91.4 | 1,092 (1,005-1,104) | 71.1 | 1,419 (1,207-1,472) | | 54.0 | 1,475 (1,123-1,623) |
|  | Ma'anshan | 79.3 | 419 (370-429) | 63.7 | 536 (437-567) | | 55.7 | 586 (452-639) |
|  | Tongling | 81.9 | 314 (268-319) | 72.3 | 414 (355-429) | | 64.9 | 467 (356-484) |
|  | Wuhu | 87.0 | 716 (242-480) | 70.0 | 931 (788-969) | | 59.2 | 1,021 (397-775) |
|  | Suzhou | 99.3 | 1,223 (927-1,202) | 84.3 | 1,627 (1,465-1,655) | | 62.7 | 1,768 (1,172-1,768) |
|  | Xuancheng | 72.6 | 504 (501-527) | 62.2 | 649 (525-690) | | 56.3 | 720 (655-813) |
| Total |  |  | 38,296 (32,781-40,071) |  | 45,694 (37,095-49,282) | |  | 53,270 (40,365-58,281) |

**Table S3.** Averaged PM_2.5_ concentrations (μg/m^3^) and PM_2.5_-related premature morality by city during pre-lockdown, level I, and level II periods in 2019

| Province | City | Pre-lockdown | | | Level Ⅰ | | Level Ⅱ | |
| --- | --- | --- | --- | --- | --- | --- | --- | --- |
|  |  | PM_2.5_ | premature mortality | PM_2.5_ | | premature mortality | PM_2.5_ | premature mortality |
|  |  |  |  |  |  |  |  |  |
| Shanghai | | 56.2 | 3,329 (2,578-3,624) | | 36.2 | 2,687 (1,707-3,385) | 27.5 | 908 (513-1,274) |
| Zhejiang | Hangzhou | 46.3 | 987 (700-1,137) | | 29.6 | 466 (272-635) | 27.6 | 323 (183-453) |
|  | Huzhou | 41.7 | 309 (209-369) | | 27.8 | 105 (59-146) | 23.3 | - |
|  | Jiaxing | 47.8 | 481 (347-549) | | 31.3 | 285 (170-379) | 24.8 | - |
|  | Jinhua | 34.3 | 366 (227-470) | | 26.4 | 92 (51-131) | 25.7 | 58 (32-84) |
|  | Lishui | 28.2 | 88 (50-123) | | 19.2 | - | 21.7 | - |
|  | Ningbo | 40.0 | 651 (433-790) | | 26.5 | 132 (74-188) | 20.7 | - |
|  | Quzhou | 33.1 | 186 (114-243) | | 23.5 | - | 27.5 | 106 (60-149) |
|  | Shaoxing | 39.8 | 483 (320-587) | | 28.3 | 207 (118-286) | 25.7 | 58 (32-84) |
|  | Taizhou | 39.9 | 665 (442-807) | | 26.4 | 131 (73-186) | 25.8 | 86 (47-124) |
|  | Wenzhou | 34.1 | 648 (401-835) | | 21.2 | - | 24.8 | - |
|  | Zhoushan | 28.3 | 33 (19-46) | | 21.7 | - | 15.7 | - |
| Jiangsu | Changzhou | 77.9 | 905 (795-928) | | 42.8 | 870 (596-1,030) | 39.0 | 883 (580-1,081) |
|  | Huaian | 81.6 | 1,000 (891-1,020) | | 54.3 | 1,180 (900-1,296) | 41.2 | 1,054 (710-1,264) |
|  | Lianyungang | 73.8 | 858 (740-886) | | 51.2 | 992 (737-1,107) | 32.7 | 561 (341-735) |
|  | Nanjing | 62.0 | 1,506 (1,216-1,602) | | 36.8 | 1,204 (770-1,507) | 30.7 | 813 (481-1,092) |
|  | Nantong | 64.9 | 1,404 (1,154-1,480) | | 38.9 | 1,227 (805-1,504) | 30.9 | 762 (452-1,021) |
|  | Suzhou | 65.6 | 2,119 (1,749-2,229) | | 36.2 | 1,606 (1,021-2,023) | 33.0 | 1,459 (890-1,906) |
|  | Taizhou | 69.8 | 904 (764-940) | | 43.9 | 917 (636-1,075) | 33.9 | 662 (409-856) |
|  | Wuxi | 61.7 | 1,182 (952-1,258) | | 33.8 | 774 (477-1,001) | 30.0 | 575 (337-779) |
|  | Suqian | 93.4 | 993 (919-1,003) | | 62.9 | 1,243 (1,009-1,318) | 45.2 | 1,157 (813-1,345) |
|  | Xuzhou | 107.9 | 1,773 (1,691-1,781) | | 65.1 | 2,229 (1,835-2,348) | 49.3 | 2,226 (1,627-2,514) |
|  | Yancheng | 63.6 | 1,338 (1,091-1,416) | | 42.0 | 1,320 (897-1,573) | 32.2 | 862 (520-1,136) |
|  | Yangzhou | 78.4 | 883 (778-905) | | 40.4 | 784 (523-947) | 31.6 | 503 (301-668) |
|  | Zhenjiang | 73.9 | 638 (551-659) | | 44.8 | 653 (457-761) | 38.0 | 599 (389-741) |
| Anhui | Anqing | 61.0 | 921 (738-983) | | 41.2 | 899 (605-1,078) | 40.1 | 1,005 (669-1,217) |
|  | Bengbu | 88.6 | 706 (644-715) | | 53.8 | 821 (624-904) | 43.5 | 792 (547-932) |
|  | Bozhou | 112.4 | 1,220 (1,171-1,224) | | 78.1 | 1,599 (1,406-1,639) | 51.7 | 1,587 (1,185-1,767) |
|  | Chizhou | 55.4 | 264 (203-288) | | 35.4 | 204 (128-259) | 33.6 | 206 (127-268) |
|  | Chuzhou | 73.1 | 818 (703-845) | | 47.9 | 897 (647-1,022) | 39.9 | 837 (555-1,016) |
|  | Fuyang | 110.6 | 2,005 (1,920-2,012) | | 71.1 | 2,581 (2,197-2,678) | 52.9 | 2,647 (1,996-2,929) |
|  | Hefei | 67.4 | 1,399 (1,167-1,465) | | 40.5 | 1,288 (861-1,555) | 36.0 | 1,207 (765-1,523) |
|  | Huaibei | 113.3 | 407 (391-408) | | 64.7 | 510 (419-537) | 52.5 | 535 (402-593) |
|  | Huainan | 92.6 | 724 (668-731) | | 59.5 | 886 (703-951) | 50.4 | 932 (688-1,046) |
|  | Huangshan | 29.8 | 75 (44-103) | | 20.5 | - | 25.4 | 10 (6-15) |
|  | Luan | 70.2 | 1,049 (889-1,091) | | 43.5 | 1,052 (726-1,238) | 41.2 | 1,139 (767-1,366) |
|  | Ma'anshan | 73.2 | 413 (355-427) | | 44.1 | 417 (290-488) | 38.2 | 392 (255-484) |
|  | Tongling | 58.5 | 286 (225-308) | | 42.0 | 293 (199-349) | 38.7 | 300 (196-368) |
|  | Wuhu | 70.5 | 692 (587-719) | | 43.3 | 689 (474-812) | 37.0 | 626 (402-781) |
|  | Suzhou | 105.1 | 1,226 (1,164-1,232) | | 61.8 | 1,514 (1,220-1,611) | 46.1 | 1,449 (1,027-1,672) |
|  | Xuancheng | 61.8 | 480 (387-511) | | 37.7 | 404 (262-501) | 35.7 | 418 (264-530) |
| Total |  |  | 36,417 (30,387-38,749) | |  | 33,158 (23,948-38,448) |  | 27,737 (18,568-33,813) |

**Table S4.** Averaged PM_2.5_ concentrations (μg/m^3^) and PM_2.5_-related premature morality by city during pre-lockdown, level I, and level II periods in 202

| Province | City | Level Ⅰ | | | | | | Level Ⅱ | | | | | |
| --- | --- | --- | --- | --- | --- | --- | --- | --- | --- | --- | --- | --- | --- |
|  |  | COPD | IHD | ALRI | Stroke | LC | Total | COPD | IHD | ALRI | Stroke | LC | Total |
| Shanghai | | 336 | 650 | 3 | 783 | 159 | 1,932 | 667 | 1,140 | 5 | 1,374 | 316 | 3,502 |
| Zhejiang | Hangzhou | 174 | 307 | 1 | 370 | 82 | 934 | 158 | 262 | 1 | 315 | 75 | 811 |
|  | Huzhou | 59 | 100 | 0 | 121 | 28 | 309 | 45 | 70 | 0 | 84 | 21 | 221 |
|  | Jiaxing | 69 | 124 | 1 | 150 | 33 | 377 | 114 | 186 | 1 | 224 | 54 | 579 |
|  | Jinhua | 133 | 226 | 1 | 272 | 63 | 696 | 117 | 190 | 1 | 229 | 55 | 592 |
|  | Lishui | 41 | 64 | 0 | 77 | 19 | 201 | 70 | 112 | 1 | 135 | 33 | 351 |
|  | Ningbo | 178 | 304 | 1 | 366 | 84 | 934 | 172 | 278 | 1 | 335 | 81 | 869 |
|  | Quzhou | 54 | 86 | 0 | 104 | 26 | 271 | 43 | 70 | 0 | 84 | 20 | 217 |
|  | Shaoxing | 116 | 202 | 1 | 243 | 55 | 616 | 157 | 262 | 1 | 316 | 74 | 811 |
|  | Taizhou | 163 | 273 | 1 | 330 | 77 | 845 | 196 | 326 | 1 | 393 | 93 | 1,010 |
|  | Wenzhou | 222 | 361 | 2 | 435 | 105 | 1,124 | 315 | 526 | 2 | 634 | 149 | 1,628 |
|  | Zhoushan | 16 | 25 | 0 | 30 | 8 | 79 | - | - | - | - | - | - |
| Jiangsu | Changzhou | 53 | 111 | 0 | 134 | 25 | 324 | 50 | 95 | 0 | 115 | 24 | 284 |
|  | Huaian | 27 | 68 | 0 | 81 | 13 | 189 | 65 | 132 | 0 | 159 | 31 | 387 |
|  | Lianyungang | 28 | 67 | 0 | 80 | 13 | 188 | 87 | 156 | 1 | 188 | 41 | 473 |
|  | Nanjing | 134 | 260 | 1 | 313 | 64 | 772 | 227 | 404 | 2 | 487 | 108 | 1,227 |
|  | Nantong | 117 | 238 | 1 | 287 | 55 | 699 | 232 | 420 | 2 | 507 | 110 | 1,270 |
|  | Suzhou | 195 | 376 | 1 | 453 | 92 | 1,118 | 278 | 512 | 2 | 617 | 132 | 1,542 |
|  | Taizhou | 54 | 117 | 0 | 142 | 25 | 338 | 125 | 238 | 1 | 287 | 59 | 710 |
|  | Wuxi | 132 | 248 | 1 | 298 | 63 | 742 | 195 | 345 | 1 | 416 | 92 | 1,048 |
|  | Suqian | 14 | 38 | 0 | 45 | 6 | 103 | 56 | 122 | 0 | 147 | 26 | 352 |
|  | Xuzhou | 21 | 59 | 0 | 72 | 10 | 162 | 75 | 175 | 1 | 211 | 36 | 497 |
|  | Yancheng | 91 | 194 | 1 | 234 | 43 | 563 | 205 | 380 | 2 | 458 | 97 | 1,141 |
|  | Yangzhou | 62 | 129 | 0 | 155 | 30 | 376 | 129 | 236 | 1 | 284 | 61 | 711 |
|  | Zhenjiang | 32 | 68 | 0 | 82 | 15 | 197 | 58 | 113 | 0 | 136 | 27 | 335 |
| Anhui | Anqing | 56 | 114 | 0 | 137 | 27 | 334 | 48 | 92 | 0 | 111 | 23 | 275 |
|  | Bengbu | 19 | 46 | 0 | 56 | 9 | 130 | 32 | 65 | 0 | 78 | 15 | 189 |
|  | Bozhou | 5 | 18 | 0 | 21 | 3 | 47 | 40 | 95 | 0 | 114 | 19 | 269 |
|  | Chizhou | 22 | 40 | 0 | 48 | 10 | 121 | 19 | 33 | 0 | 39 | 9 | 100 |
|  | Chuzhou | 33 | 74 | 0 | 89 | 16 | 212 | 49 | 94 | 0 | 114 | 23 | 280 |
|  | Fuyang | 15 | 46 | 0 | 55 | 7 | 123 | 52 | 120 | 0 | 145 | 25 | 343 |
|  | Hefei | 106 | 222 | 1 | 267 | 50 | 646 | 168 | 330 | 1 | 398 | 79 | 976 |
|  | Huaibei | 5 | 14 | 0 | 16 | 2 | 37 | 10 | 23 | 0 | 28 | 5 | 67 |
|  | Huainan | 13 | 34 | 0 | 41 | 6 | 94 | 22 | 48 | 0 | 58 | 10 | 138 |
|  | Huangshan | 20 | 30 | 0 | 37 | 9 | 96 | 34 | 54 | 0 | 65 | 16 | 169 |
|  | Luan | 56 | 118 | 0 | 142 | 26 | 343 | 76 | 154 | 1 | 186 | 36 | 452 |
|  | Ma'anshan | 20 | 43 | 0 | 52 | 10 | 125 | 20 | 38 | 0 | 45 | 10 | 113 |
|  | Tongling | 17 | 35 | 0 | 42 | 8 | 101 | 24 | 47 | 0 | 57 | 11 | 140 |
|  | Wuhu | 38 | 81 | 0 | 98 | 18 | 236 | 67 | 131 | 1 | 158 | 32 | 388 |
|  | Suzhou | 18 | 48 | 0 | 58 | 8 | 132 | 62 | 135 | 0 | 163 | 29 | 390 |
|  | Xuancheng | 38 | 74 | 0 | 89 | 18 | 218 | 50 | 94 | 0 | 114 | 24 | 282 |
| Total | | 3,004 | 5,729 | 23 | 6,907 | 1,422 | 17,085 | 4,609 | 8,302 | 35 | 10,009 | 2,181 | 25,136 |

**Table S5**. Estimated number of avoided premature mortality during Level I and Level II periods
